# Innate Immune Recognition: An Issue More Complex Than Expected

**DOI:** 10.3389/fcimb.2019.00241

**Published:** 2019-07-03

**Authors:** Klara Kubelkova, Ales Macela

**Affiliations:** Department of Molecular Pathology and Biology, Faculty of Military Health Sciences, University of Defence, Hradec Kralove, Czechia

**Keywords:** innate immune recognition, signaling pathways, phagocytosis, intracellular replication, *Francisella tularensis*

## Abstract

Primary interaction of an intracellular bacterium with its host cell is initiated by activation of multiple signaling pathways in response to bacterium recognition itself or as cellular responses to stress induced by the bacterium. The leading molecules in these processes are cell surface membrane receptors as well as cytosolic pattern recognition receptors recognizing pathogen-associated molecular patterns or damage-associated molecular patterns induced by the invading bacterium. In this review, we demonstrate possible sequences of events leading to recognition of *Francisella tularensis*, present findings on known mechanisms for manipulating cell responses to protect *Francisella* from being killed, and discuss newly published data from the perspective of early stages of host–pathogen interaction.

## Introduction

Innate immune response constitutes the first line of defense against bacterial infections. The dominant role in triggering and streamlining innate immunity is played by the innate immune recognition process in combination with the intrinsic characteristics of the microorganism and its host. The general basic assumption regarding immunity against microbes is that the response is based on the mechanisms of recognition and, equally important, on self-non-self discrimination. The basic milestones for understanding immunity as a phenomenon of defense were defined in the 1950s. Theories on clonal selection or actively acquired tolerance have defined the scope of adaptive immunity. Up to the early 1990s, immunologists were focused on the mechanisms of adaptive immune recognition, including the structure and function of the antigen receptors, the mechanisms of major histocompatibility complex restriction, and the intercellular communication controlling the final immune responsiveness. Little was known, however, about the processes that lead to the activation of adaptive immune responses. Constituting an important milestone in this respect is a conceptual framework of the innate immune recognition implemented by a limited number of germline-encoded receptors that was proposed by Janeway (1989). His pattern recognition theory described how the process of innate immune recognition allows the immune system to distinguish its non-infectious self from infectious non-self.

Recently, the toll-like receptors, homologs of the *Drosophila* toll protein, have been regarded as the basic pattern recognition receptors that, after ligation, generate the signals that initiate the activation of adaptive immunity (Kopp and Medzhitov, 1999; Modlin et al., 1999; Möller, 1999; Muzio and Mantovani, 2000). Toll-like receptors (TLRs) play a dominant role in pathogen recognition and initiation of inflammation and immune responses (Kawai and Akira, 2009; Kumar et al., 2009). Stimulation of TLRs by microbial products leads to the activation of signaling pathways that result in the expression of antimicrobial genes and inflammatory cytokines. In addition, stimulation of TLRs triggers dendritic cell maturation and results in the induction of costimulatory molecules and increased antigen-presenting capacity (Granucci et al., 2005; Dowling and Mansell, 2016). Thus, microbial recognition by TLRs helps to direct adaptive immune responses to antigens derived from microbial pathogens. TLRs are not, however, the only receptors ensuring the innate immune recognition. The group of complement receptors, scavenger receptors, and C-type lectin receptors (including Dectin-1) all are receptors known as pattern recognition receptors (PRRs). These are receptors that, for example, recognize the pathogen-associated molecular patterns (PAMPs) (Gordon, 2002) and, to some extent, determine the fate of infected cells (Franz and Kagan, 2017). Then there is the still-growing family of membrane as well as secreted molecules that ensure recognition of prokaryotic cells or viruses by eukaryotic cells of multicellular organisms. An example can be seen in the peptidoglycan recognition proteins (PGRPs), which can be produced in secreted or membrane form and also rank among the PRRs (Royet et al., 2011; Dziarski et al., 2012; Zhang et al., 2012). Another example is lipopolysaccharide binding molecule (LBP), which, after binding to lipopolysaccharide (LPS), interacts with CD14, the co-receptor for TLR4 (Kitchens, 2000; Tapping and Tobias, 2000; Rosadini and Kagan, 2017). Which receptors are responsible for the innate immune recognition of a given microorganism at the cell membrane depends upon the microorganism itself, the host cell type and its expressed membrane receptors, and the conditions under which the host–pathogen interaction is realized.

Innate immune recognition is not realized solely at the cell membrane, however. If the cell ingests a microbe by the process of phagocytosis or macropinocytosis, the recognition continues at a phagosome vacuole where such PRRs as TLR 3, TLR 7/8, TLR 9, and TLR 13 and/or C receptors and Fcγ receptors are expressed and can sense the microbe *per se* and/or the products of enzymatic microbial disintegration (Tjelle et al., 2000; García-García and Rosales, 2002; Swanson and Hoppe, 2004; Moretti and Blander, 2014). If microbes have the ability to survive the intraphagosomal milieu and escape into the cytosol, then other cytosolic recognition systems are available. The retinoic acid-inducible gene-I (RIG-I)-like receptors (RLRs), nucleotide-binding oligomerization domain, leucine-rich repeat-containing protein receptors (NLRs), the family of absent in melanoma (AIM)-like receptors (ALRs), along with a number of cytosolic DNA sensors are at the cell's disposal for intracytosolic recognition of conserved structures of microorganisms (Franchi et al., 2009; Muñoz-Wolf and Lavelle, 2016). Ligation of all these sensors is critical for inducing innate immune defense. One of the critical steps for this event is the assembly of a specific protein complex that includes NLRs or ALRs, the apoptosis-associated speck-like protein containing a C-terminal CARD adapter, and pro-caspase-1 (Lamkanfi et al., 2007; De Zoete et al., 2014). This molecular complex has been termed the inflammasome and constitutes one of the oligo- or multi-molecular complexes in the cytosol. Similar complexes are myddosome (Deguine and Barton, 2014; Gay et al., 2014), calcium signalosome (Filippi-Chiela et al., 2016), and apoptosome (Riedl and Salvesen, 2007) and are protein complexes ensuring the functional realization of receptor signal messages.

## Innate Immune Recognition of Intracellular Bacteria: *Francisella tularensis* as a Model

Innate immune recognition is a process that initiates the basic cellular responses to mutual interaction of the host cells with the invading microbes. Moreover, the innate immune recognition of PAMPs activates the innate immune responses, which is a prerequisite step needed for generation of immunogenic signals inducing one of the adaptive immunity's specific arms.

### *Francisella tularensis* as a Model

*Francisella tularensis* (*F. tularensis*) has been used frequently, along with *Listeria monocytogenes*, or *Salmonella typhimurium*, as a model of bacteria that survive an intraphagosomal milieu inside the cells of the mononuclear phagocytic system. Moreover, *Francisella* species, similarly as do *Listeria* species, escape from the phagosome and proliferate in the cytosol. An advantage of *Francisella* models for the study of host–pathogen interactions consists in the genus's four species that are currently recognized: *F. endosymbionts, F. philomiragia, F. novicida*, and *F. tularensis*, the latter having the three subspecies *tularensis, holarctica*, and *mediasiatica* (Duncan et al., 2013). Recently developed classification methods, however, have enabled the reclassification of *Wolbachia persica* to *F. persica* (Larson et al., 2016) and identification of the new members of the *Francisella* genus *F. frigiditurris* (CA97-1460), *F. opportunistica* (MA06-7296), *F. salina* (TX07-7308), and *F. uliginis* (TX07-7310) (Challacombe et al., 2017a,b). Moreover, the interaction of *Francisella* with the host cells has some specific features that make this microorganism a unique model. For example, LPS, with its atypical lipid A, fails to substantially activate TLR4, which is a unique characteristic among Gram-negative bacteria (Okan and Kasper, 2013; Robert et al., 2017). Encapsulation makes this microbe invisible for recognition by IgM and C3 and endows the bacterium with serum resistance (Brock and Parmely, 2017). The specific features include also the Type VI-like secretion system (T6SS) of *Francisella* species. Various proteins are reported to be secreted by *Francisella*, but the mechanisms for their secretion remain unknown. *Francisella* has the *Francisella* pathogenicity island (FPI)-encoded Type VI-like secretion system (Spidlova and Stulik, 2017; Clemens et al., 2018), but its function has not yet been reported. Also, the exact functions of Type IV pili have not been satisfactorily clarified. The existence of *Francisella* genes for exotoxin(s) or gene clusters encoding type III, type IV, or type V secretion systems have never been confirmed (Larsson et al., 2005). One of the recently very popular possible explanations for how protein secretion occurs is that it can be through production of outer membrane vesicles, which, moreover, have a specific shape (McCaig et al., 2013; Chen et al., 2017; Stevenson et al., 2018) and can contribute to (or interfere with) the innate immune recognition of the pathogen.

The majority of tularemia cases in humans are caused by *F. tularensis* subsp. *tularensis*, which is found exclusively in North America, and by *F. tularensis* subsp. *holarctica*, which is found throughout the northern hemisphere. *F. tularensis* subspecies *tularensis* and *holarctica* are highly virulent for humans and many other mammalian species, even as other strains are less virulent (Tärnvik and Berglund, 2003). *Francisella* infects invertebrates as well as vertebrates. Neutrophils, macrophages, dendritic cells, B cells, hepatocytes, endo/epithelial cells, and fibroblasts constitute the target cells for interactions in the contexts of vertebrate hosts (Sjöstedt et al., 1996; Krocova et al., 2008).

### Cell-Surface Recognition of *Francisella*—A Challenge for Innate Immune Receptors

Knowing there to be multiple cell types (subtypes) expressing multiple functionally divergent receptors interacting with the microbes in specific microenvironments, it is quite logical to assume there must be multiple possibilities for affecting innate immune recognition. The basic assumption regarding the interaction of the host with microbes must be considered. In this respect, there are different requirements for different cell (sub)types to interact with the microbes. Uptake of *Francisellae* by neutrophils and dendritic cells is dependent on opsonization (Proctor et al., 1975; Ben Nasr et al., 2006), while *Francisella* entry into macrophages is thought to be both opsonin-dependent and independent (Clemens et al., 2005; Balagopal et al., 2006). Thus, the requirements for realizing the interaction are clearly dependent upon expression of the cell surface receptors of the individual cell involved in the primary interaction with the microbe. The list of cell surface receptors that have been identified as important for the interaction with *Francisellae* contains the TLR chains of TLR2, TLR6, and, according to contradictory findings, also TLR4 (Dueñas et al., 2006; Katz et al., 2006; Li et al., 2006; Cole et al., 2007; Abplanalp et al., 2009); complement receptors (Balagopal et al., 2006; Ben Nasr et al., 2006; Geier and Celli, 2011; Schwartz et al., 2012; Plzakova et al., 2015); Fc gamma receptors (Balagopal et al., 2006; Plzakova et al., 2015); mannose receptors (Schulert and Allen, 2006); class A scavenger receptor (Pierini, 2006); and finally cell surface exposed nucleolin (Barel et al., 2008, 2010; Barel and Charbit, 2014). Uptake of unopsonized *Francisella* depends largely, but not exclusively, upon the mannose receptor with the consequence of rapid escape from the phagosome and massive proliferation in cytosol (Balagopal et al., 2006; Schulert and Allen, 2006). Uptake of serum-opsonized *Francisellae* is rather an event realized by several receptors and followed by delayed phagosome escape. Simply stated, the cell surfaces of both *Francisella* and host cell at the time of interaction together dictate the profile of the host cell–pathogen interaction and subsequently the type of induced mechanisms of immune response.

**Figure 1:**
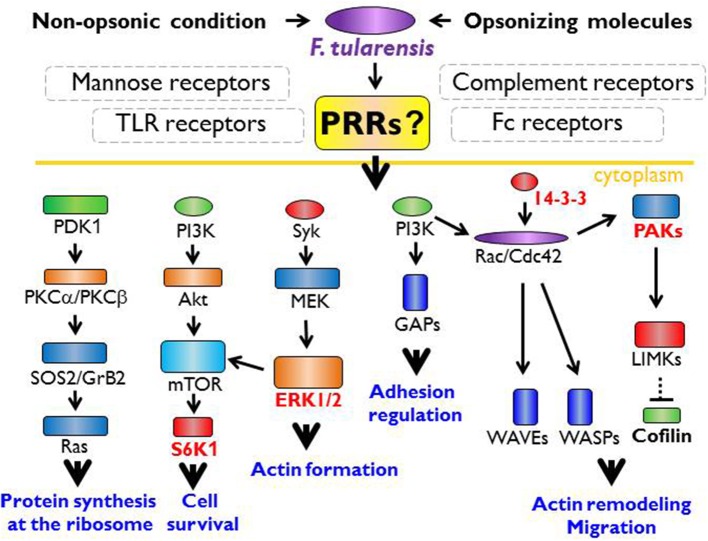
The innate immune receptor engagement and possible signaling pathways in actin cytoskeleton remodeling. Within the common paradigm of innate immune recognition, there is no doubt the first signal for recognition of *Francisella* spp. must originate from the cell surface receptors. *Francisella* is recognized via TLR (Toll like receptor) heterodimers, especially TLR1 or 6, TLR2, and TLR4 that lead to myddosome formation followed by activation of NFκB (nuclear factor kappa-light-chain-enhancer of activated B cells), p38 protein and the activation of inflammatory cytokine genes. The signaling through TLR2 and TLR4 is possible only from the phagosome. Thus, TLR-MyD88 (myeloid differentiation primary response 88) complex is moved to the lipid rafts followed by the endocytosis. Nevertheless, there should be other signal from unknown receptor that ensures the moving of TLR-MyD88 complex into the phagosome by activation of actin cytoskeleton remodeling processes. From published data, the signaling pathways via S6K1 (ribosomal protein S6 kinase beta-1), ERK1/2 (extracellular signal-regulated kinase 1), 14-3-3 protein, or PAKs (p21-activated kinases) seem to be important for the actin remodeling, the actin cytoskeleton activation, and the regulation of transcription/translation needed for signal transduction and functional restructuralization.

It is generally accepted that the innate immune recognition of *Francisella* with its TLR2 ligands is by TLR2/TLR1 or TLR2/TLR6 heterodimers, which are associated with the activation of MyD88-dependent signaling pathways and myddosome formation (Figure 1). This process plays a critical role in the induction of innate immune responses to *Francisella* (Collazo et al., 2006; Cole et al., 2007; Russo et al., 2013), whereas TLR2 engagement during the induction of adaptive immune responses is not required (Roberts et al., 2014). Activation of myddosome formation initiates subsequently the NF-κB signaling pathway and pro-inflammatory cytokine production. But, in parallel, there is data indicating that the control of *F. tularensis* infection in tissues is dependent upon the activation of MyD88 signaling only in hematopoietic cells and not in myeloid and dendritic cells (Skyberg and Lacey, 2017). The TLRs-mediated signaling thus seems, under some circumstances, to be of secondary importance or, alternatively, is inhibited or modulated by signals originated from *Francisella* metabolic activities inside a cell. Thus, survival or programmed cell death of the infected cell may be dictated by modulation of signaling pathways by invading *Francisellae* (see below). The data from *in vitro* systems has demonstrated that *Francisella* activates multiple signaling pathways (Rajaram et al., 2009; Edwards et al., 2013; Fabrik et al., 2018), among these being Akt, ERK, Rac/Cdc42, JNK/c-Jun, and/or p38 signaling modules (Clemens and Horwitz, 2007; Rajaram et al., 2009; Edwards et al., 2013; Fabrik et al., 2018). Moreover, the signals are differentially initiated by virulent and attenuated strains and are realized in temporally separate phases (Fabrik et al., 2018).

While some signaling pathways have already been identified, the receptors from which these signaling pathways are activated are more likely to be only suspected. One of the reasons for this is that a number of receptors share the same adaptor protein(s) that can activate the specific signaling pathway. One example is MyD88, which is regarded as a canonical adaptor for inflammatory signaling pathways. MyD88 links IL-1 receptor (IL-1R) or TLR family members to interleukin-1 receptor associated kinase family kinases, thereby creating a multimeric complex reacting with a member of the TNF receptor associated factor protein family. This complex is known as a myddosome. The MyD88 downstream signaling components activation depends upon the context of signaling initiation, the cell (sub)type, and the microenvironment of signal initiation. Such stringent specification of initial conditions for signal initiation can lead to distinct outputs of receptor(s) ligation(s) (Deguine and Barton, 2014). The ligation of more than one type of PRR may have an enhancing or debilitating effect on downstream signaling leading to expression of target genes. An example can be seen in the crosstalk between complement and TLR signaling pathways, as has repeatedly been demonstrated in numerous model systems (Hajishengallis and Lambris, 2010, 2016). Another reason why there are doubts in identifying the signaling receptor can be overlapping and networking of signaling pathways leading from different receptors, as is the case with mitogen activated protein kinases (MAPKs). Many cytokines are released from the cell immediately (in contrast to IL-1β, which requires the additional step of post-translational processing) and can constitute the signals initiating the epigenetic reprogramming of bystander cells and, in such manner, modulating the recognition processes. These facts need to be taken into account especially when analyzing the results of *in vitro* studies. For example, *F. tularensis* strain LVS induces the production at substantial levels of TNF-α within 60 min after infection of various human as well as murine monocyte/macrophage cells (Telepnev et al., 2005). Thus, the demonstrated activation of such signaling modules as ASK1/p38/MAX (Telepnev et al., 2005; Fabrik et al., 2018), JNK/c-JUN (Telepnev et al., 2005), Ras/PKCα/PKCβI (Akimana et al., 2010), Raf/MEK(s)/ERK (Parsa et al., 2008; Fabrik et al., 2018), and Akt/SHIP/PKB (Rajaram et al., 2006, 2009) may be mediated directly through TLRs ligation or indirectly by the effects of cytokines produced early and operating in an autocrine or paracrine manner (Akira, 2003; Walsh et al., 2015).

### Complexity of Innate Immune Recognition in Terms of Spatial Distribution of Cellular Targets

The activity of early produced cytokines may be significant for bystander cell(s) of the same or different subtype(s) that will recognize the bacterium in the second order. The response to early produced cytokines is another complicating factor when determining the initiating receptor type. Cells responding to early produced cytokines, already just epigenetically reprogrammed, may use (a) different receptor(s) and activate different signaling pathway(s) than do those cells originally recognizing a bacterium. Such a situation certainly exists in both *in vitro* and *in vivo* infection models. Concerning *in vitro* systems, their design and implementation for host–pathogen interaction studies (i.e., infection biology studies) have some specific features that must be respected when interpreting the results: (a) In addition to living microbes, the infection dose contains a certain number of dead microorganisms and, possibly, some components of microbial bodies after their disintegration. (b) Not all cells in culture are infected at the time zero (or the same time after opsonization of microbial load). (c) Even after repeated and thorough washing of an infected cell culture there still remains a certain proportion of microorganisms in the supernatant. (d) Microbe-free cell–cell interactions among cells of a culture cannot be excluded. (e) The cells within a cell culture react to the cell culture microenvironment and respond to this regardless of the contact with the model microorganism. Moreover, when using an *in vitro* study, we resign ourselves to identifying functional modules of immune responsiveness due to the total elimination of spatiotemporal changes in the microenvironment that influence the cell responsiveness. That means we obtain a rather static view as to the hierarchy of signals controlling the innate immune recognition.

We face completely different challenges when using *in vivo* studies. Intracellular bacteria are spread through the body by infected cells or freely by body fluids (Forestal et al., 2007; Bar-Haim et al., 2008; Yu et al., 2008). Moreover, dissemination of *Francisellae* that reaches the target organs can be realized by trogocytosis between bystander cells (Bourdonnay and Henry, 2016; Rodriguez et al., 2016; Steele et al., 2016). Multiple mechanisms of dissemination from cell to cell provide a variety of options for cells that will be infected in secondary order to recognize *Francisella*. Moreover, these cells, as the cellular components of innate immune communication, might be differentially programmed for innate recognition of pathogens, depending on the specific microenvironment within which they happen to be located. This specific microenvironment may already have been modulated by cells infected in primary order. The dissemination of *Francisella* to internal organs (spleen, liver, lungs) requires just a matter of hours in cases of intravenous or intraperitoneal infection, but this dissemination will take a number of days in cases of intranasal or intradermal infections (Fortier et al., 1991). Such cytokines as IFNβ (Jacobs and Ignarro, 2001) or TNFα (Telepnev et al., 2005) that are produced immediately after infection or after activation of mononuclear phagocytic cells by microbial components can affect the microenvironment of those cells that will be infected in the following order. The production of IL-1β by macrophages or dendritic cells infected by *Francisella* has been demonstrated between 5 and 8 h post infection (Gavrilin et al., 2006; Li et al., 2006; Fernandes-Alnemri et al., 2010). Such timing is still sufficient to modulate the response of innate immune cells localized in distant organs to *Francisellae*. Recently, therefore, developing a complete understanding of *in vivo* pathogen innate immune recognition processes, and subsequently of innate immune intercellular communication, has become a key biological issue of infection biology.

### Primary Interaction Initiates Entry Into Host Cell

The entry of *Francisella* into the host cells has several basic features that are dependent on the host cell types. *Francisella* uptake by macrophages, which are the most studied targets of host cell–*Francisella* interaction, occurs by way of asymmetric, spacious pseudopod loops through a process that has been named looping phagocytosis (Clemens et al., 2005; Santic et al., 2006; Clemens and Horwitz, 2007). This process is probably related (if not identical) to macropinocytosis, which is achieved by actin filament-driven asymmetric plasma membrane protrusions (Kerr and Teasdale, 2009). The unique feature of looping phagocytosis is recruitment of cholesterol-rich lipid rafts with caveolin-1 enabling successful entry into macrophages (Tamilselvam and Daefler, 2008). The invasion of *Francisella* into non-professional phagocytic cells occurs in association with membrane cholesterol-rich lipid domains and is dependent on clathrin, not caveolin-1 (Law et al., 2011), and it seems to be related to macropinocytosis (Lindemann et al., 2007; Craven et al., 2008; Bradburne et al., 2013). Like macropinocytosis, looping phagocytosis is inhibited by microfilament and microtubule activity inhibitors, thus suggesting that both the actin and microtubule cytoskeletons are important for invasion. Overall, one can conclude that the entry of *Francisella* into host cell combines the markers of phagocytosis, micropinocytosis, and/or receptor-mediated endocytosis, and it initiates such processes as can be effective for the specific target cell (sub)type. Moreover, data documenting the indistinguishable kinetics of live and paraformaldehyde-fixed *F. tularensis* live vaccine strain association with and internalization by mouse lung epithelial cell line has provided evidence that cell invasion is mediated by a preformed ligand on the bacterial cell surface and is driven entirely by host cell processes (Craven et al., 2008).

The primary interaction of *Francisella* with the host cell starts by association of their surfaces, and it can be assumed that the signals come from receptors of the host cell outer membrane (Figure 1). This is certainly true even in the case that interaction is initiated with secreted bacterial molecules that can be produced into inter-membranous space. Moreover, it may be assumed that the first signaling wave will be oriented to the activation of cytoskeletal rearrangement ensuring internalization of the adhered bacterium. The TLRs seem to be poor candidates for this purpose due to the fact that their signaling is dependent on their MyD88- and lipid rafts-dependent translocation into phagosome (Stack et al., 2014; Park et al., 2017). This assumes the existence of signals coming from other receptor types that subsequently activate the actin remodeling processes. Inasmuch as the actin seems to be indispensable for *Francisella* internalization, the potential candidate signals for actin remodeling can be the Rac/Cdc42/PAKs signaling module complemented by 14-3-3 protein(s). The actin polymerization is associated with binding of unphosphorylated cofilin at Ser3 to actin (Moriyama et al., 1996; Bamburg, 1999). The phosphorylation and dephosphorylation of cofilin are under control of the actin-binding kinases (Edwards et al., 1999; Prunier et al., 2017) that are activated by various kinases, including PAK1, PAK2, and PAK4, or by 14-3-3 proteins (Brandwein and Wang, 2017). Initial signals originate in both cases from members of the Rho GTPase family. In parallel, engulfment of *Francisella* can be initiated also by activation of tyrosine kinase Syk and downstream effector ERK kinases (Parsa et al., 2008).

If activation of the small signaling G proteins may be the signals for *Francisella* internalization into the host cells, then activation of TLRs signaling pathways seems to be the signal for initiation of innate immune response (see Figure 1). The initiation of myddosome assembly and downstream signaling through the MAPK/ERK pathway (also known as the Ras-Raf-MEK-ERK pathway) or c-Jun terminal kinases (JNKs) signaling, which kinases are responsive to stress stimuli, can create, along with activation of the Rho GTPase family proteins, the first batch of signals allowing to continue the spatiotemporal process of innate immune recognition. Concomitant activation of signals for *Francisella* internalization and induction of intercellular innate immune communication make it difficult to identify and understand the innate immune recognition and response of cells in distant target organs to interaction with *Francisella*. Such response, as well as intracellular trafficking of *Francisella* in these cells, may be dissimilar from the response of cells infected at the periphery.

### *Francisella* Inside the Phagosome

Closure of phagosome and initiation of intracellular trafficking are processes not yet fully understood. On the one hand, *Francisella* is in contact with receptors with which it has been interacting on the surface of the host cell. On the other hand, the adaptation of bacteria to a new environment can change the molecular relationships of mutual host–bacteria interaction by expressing different PAMPs. Thus, different PAMPs need different specific receptors to detect the presence of microbes and their products (Medzhitov, 2009). In this respect, one should resist taking the rather dogmatic view that the site of receptor-ligand interaction is necessarily the site of signaling. Many PRRs must translocate to a second cell compartment for signal transduction to occur. Both cell surface TLRs, which were presented as PRRs for *Francisella* (heterodimer of TLR2/TLR1 or TLR2/TLR6 and TLR4), must be mobilized into lipid rafts to induce MyD88-dependent signal transduction. Moreover, TLR4 after translocation into endosomes induces TRIF-dependent signaling (Triantafilou et al., 2002, 2006; Kagan et al., 2008; Zanoni et al., 2011). It seems likely that the general feature of PRRs signaling is spatiotemporal separation of sites for ligand binding and signal transduction, respectively. The phagosome, in some stage of its maturation, may thus be the site enabling initiation of the TLR2 signaling pathway.

The early events of *Francisella* enclosed in a *Francisella*-containing phagosome comprise transient interaction of the phagosome with early and late endosomes that is accompanied by mild acidification of the vacuolar space. The *Francisella* responds to environmental changes by expression of *Francisella* pathogenicity island (FPI) proteins (Chong et al., 2008). FPI encodes a cluster of 17 genes that is duplicated at the genomes of *F. tularensis* subsp. *tularensis* and *holarctica* (Nano et al., 2004). This cluster of genes shares homology with the genes coded bacterial type VI secretion system (T6SS) (Nano et al., 2004; Ludu et al., 2008; Bröms et al., 2009, 2010), and some of the proteins encoded by FPI are actually secreted into macrophages. Among these, VgrG is secreted into cell culture supernatants or directly into the macrophage but does not require the expression of any other FPI genes (Barker et al., 2009). This fact somewhat complicates the interpretation of genes encoded by FPI as genes coding the components of functional T6SS. The presence of VgrG contributes to secretion of other bacterial proteins, specifically Igll, into macrophages and may suggest a participation of this protein in some alternative to T6SS (Barker et al., 2009). Complications with *Francisella* T6SS functionality can be related to an unusual arrangement needed for secretion of unusual substrates that are unique for *Francisella* species. This argument can be supported by noting the secretion FPI proteins IglE, IglC, VgrG, IglI, PdpE, PdpA, IglJ, and IglF, which is dependent on the basic structural components DotU, VgrG, and IglC, as well as IglG (Bröms et al., 2012). Generally speaking, the early response of *Francisella* to intraphagosomal milieu is to upregulate general and oxidative stress response genes and the genes referred to as virulence factors, among which are the core components of T6SS and the substrates to be secreted (Wehrly et al., 2009). These early events terminate in the *Francisella* escape from the phagosome (Golovliov et al., 2003; Clemens et al., 2004; Clemens and Horwitz, 2007; Chong et al., 2008). A substantial role may be played by the *Francisella* non-canonical T6SS that seems to be critical for intracellular trafficking and proliferation of *Francisella* inside host cells (Clemens and Horwitz, 2007; Lindgren et al., 2013; Long et al., 2013; Brodmann et al., 2017;Clemens et al., 2018).

The mammalian cell hosting the *Francisella*-containing phagosome has another possibility to recognize the invading bacterium by a set of phagosomal membrane PRRs. In cases of living bacteria and/or (more likely) dead bacteria or proliferation of incompetent bacteria that can be destroyed inside the phagosome in some specific phase of phagosome maturation, these bacteria can be an object of different PAMPs delivery. Their recognition initiates signaling pathways leading to innate immune responses. The intracellular TLRs, including TLR3, TLR7, TLR8, TLR9, and murine TLR13, that are located within endosomes, are capable of detecting nucleic acids (Blasius and Beutler, 2010). The ligands for TLR3 are dominantly viral dsRNA, while those for TLR7 and TLR8 are viral ssRNA. TLR9, unlike other TLRs, recognizes dominantly bacterial and viral DNA containing unmethylated CpG DNA motifs (Pandey et al., 2014). Murine TLR 13 has been shown to recognize bacterial but not eukaryotic 23S ribosomal RNA (Hidmark et al., 2012; Li and Chen, 2012; Oldenburg et al., 2012). Recognition of intraphagosomal PAMPs by TLRs initiates a specific as well as overlapping signaling cascades depending on the cell (sub)type of the interacting host cell. With the exception of TLR3, the ligation of the TLRs leads to hemophilic binding of receptor and adapter MyD88 TIR domains. MyD88 recruits IRAK4, which initiates myddosome formation and activation downstream in NF-κB or JNK, p38MAP kinase, or CREB signaling pathways. TLR9 of plasmacytoid dendritic cells utilizes a distinct pathway characterized by formation of incomplete myddosome or engagement of osteopontin and activation of transcription factor IRF7. The TLR3 signaling of conventional dendritic cells and macrophages occurs through TRIF, TRAF6, and RIP1, a complex that associates with TRAF3 and activates TBK1 and IKK3, which, as a final event, phosphorylate IRF3. IRF3 is a potent activator of the IFNβ gene, and IRF 7 efficiently activates both IFNα and IFNβ genes (Marié et al., 1998; Sato et al., 1998a,b; Zhao et al., 2015). If we admit that DNA or ribosomal RNA of *Francisella (*does not matter whether this RNA is secreted or derived, similarly as bacterial DNA from decayed cells or from outer membrane vesicles) will be recognized by TLR9 or TLR13, respectively. This recognition event can be the source of IFNβ constituting the end point of a second batch of recognition signals. Such signaling could be one of the basic premises for final cytosolic recognition of *Francisella* realized on the single cell level.

### Cytosolic Sensing of Essential Bacterial Signature

The escape of *Francisella* into the cytoplasm plays a pivotal role in infection recognition and control (Mariathasan et al., 2005; Fernandes-Alnemri et al., 2010; Jones et al., 2010; Rathinam et al., 2010). There are at least three general processes of cell physiology, along with TLRs signaling, which contribute to the recognition of microbe-associated molecular patterns. The connection of cell-autonomous immunity, the DNA cytosolic surveillance pathways, and unfolded protein response underscore the complexity of innate immune recognition and activation of multiple signaling pathways.

Originally, it was demonstrated that the connection between type I IFN signaling and inflammasome activation (as two sequential events) is critical for recognition of *Francisella* localized in the cytosol (Henry et al., 2007; Fernandes-Alnemri et al., 2009, 2010; Jones et al., 2010). In general, the activation of inflammasomes occurs through sensing of free extraneous dsDNA. AIM2, an interferon-inducible protein which by its C-terminal HIN domain binds double-stranded DNA, was identified as the dominant cytosolic sensor recognizing *Francisella's* DNA during the early stages of host cell–*Francisella* interaction in *in vitro* systems. The AIM2-dsDNA complex initiates the oligomerization of AIM2 with ASC, and this complex consequently attracts procaspase 1 (Lugrin and Martinon, 2018).

Thus, the essential prerequisite for the function of inflammasome is the release of bacterial DNA into the cytosol. This may occur either within the phagosome by disruption of some part of the bacterial load or in the cytosol directly. In both cases, disruption of bacterial integrity is needed. Recently, it has come to appear likely that the critical event needed for release of bacterial DNA into host cell cytosol is induction of the members of the IFNs family. This is because they are engaged in expression of the large family of interferon-inducible GTPases that includes both the immunity-related GTPases (IRGBs) and guanylate-binding proteins (GBPs) (Meunier and Broz, 2016). GBPs and IRGs function in a cell-autonomous immunity and have been shown to target both vacuolar and cytosolic pathogens by destruction of vacuolar and/or pathogen membranes (Man et al., 2015; Meunier et al., 2015). In the framework of *Francisella* models, the GBPs, specifically GBP2 and GBP5, if produced, are recruited onto cytosolic *Francisella* and are targets for attracted IRGB10 (Meunier et al., 2015; Man et al., 2016). The result of IRGB10 targeting to *Francisella* is the disruption of bacterial integrity and liberation of bacterial DNA, which then becomes accessible to recognition by cytosolic DNA sensors.

It should be noted that AIM2 is not the sole inflammasome sensor recognizing *Francisella* localized inside the host cell cytosol. Despite data from a mice model showing that other cytosolic DNA sensors including NLRP1, NLRP3, NLRC4 or other known NLRs are not involved in *Francisella* sensing (Fernandes-Alnemri et al., 2010), there are other data documenting that, under some specific situations, the molecular components of *Francisella* are recognized by NLRP3 sensor. IRGB10 has been shown to be recruited onto the bacterial surface, where, together with GBPs, it enables breakdown of the bacteria and exposure of bacterial DNA to the cytosolic sensors. Similarly, this may expose the LPS on fragments of the bacterial membrane to recognition by LPS-sensing caspase-11 (murine homolog of human caspase 4) and in this manner activate NLRP3 inflammasome by a non-canonical pathway (Gavrilin and Wewers, 2011; Vanaja et al., 2015; Man et al., 2016).

Older data from murine models had demonstrated the activation of NLRP3 by heat-killed *F. novicida* to be dependent on pannexin 1 when ATP is provided (Kanneganti et al., 2007). Also, crosstalk between the Fc gamma receptors and TLR2 initiated by *Francisella* inactivated by opsonizing antibodies is shown to lead to NLRP3 inflammasome-dependent IL-1β production (Duffy et al., 2016). Apart from that, human NLRP3 has been shown to be sufficient to mediate inflammasome-dependent production of IL-1β in response to *Francisella* in both epithelial and monocyte/macrophage cell lines (Atianand et al., 2011). These data contradict the general beliefs that caspase 11 is a sensor of penta- and hexa-acylated lipid A (Hagar et al., 2013) and that the *Francisella* species have only tetra-acyl lipid A, which is not recognized by TLR4 (Phillips et al., 2004; Hajjar et al., 2006; Schilling et al., 2007). Thus, the engagement of NLRP3 in *Francisella* recognition may be the result of combined (oligo)multistep PAMPs-DAMPs (damage-associated molecular patterns) activation events, as can be deduced from the chemical spectrum and structural diversity of NLRP3-activating stimuli (He et al., 2016).

The key pathway to recognition of extraneous DNA in the cytosol is the signal transduction cascade cGAS/STING/TBK1/IRF3 that triggers a type I IFN transcriptional response (Xia et al., 2016). If recognition signals occurring at the phagosome stage of *Francisella* intracellular trafficking and leading to type I IFN production indeed exist, this means that the cGAS/STING/TBK1/IRF3 signaling cascade replaces or complements (reinforces or intensifies) the recognition signals from the phagosome. The cGAS, a cyclic GMP-AMP synthase, is a cytosolic DNA sensor activating the type I interferon pathway. cGAS binds to microbial DNA and catalyzes the synthesis of cGAMP, which functions as a second messenger in STING activation (Tao et al., 2016; Xia et al., 2016). As an initiator of molecular signal, cGAS has been shown to be required for production of type I IFN in *Francisella* infection of murine macrophages. For effective type I IFN response, surprisingly, signaling by the two DNA sensors cGAS and Ifi204 is needed (Storek et al., 2015). How these two sensors cooperate during the process of bacterial DNA recognition and activation of type I IFN transcription can be extrapolated from data concerning IFI16, which is a human ortholog of IFI204 (Zhao et al., 2015). Published data indicate that IFI16 operates in a two-step module: First, IFI16 enhances cGAS-mediated cGAMP production (probably due to higher affinity for dsDNA than cGAS, or to appropriate subcellular localization) and, in parallel, it recruits TBK1 to STING and forms a signaling complex with STING and TBK1. This allows TBK1 to phosphorylate STING and activate the STING signalosome (Jønsson et al., 2017). A similar study published almost at the same time as that cited immediately above indicates that IFI16 is required not only for STING phosphorylation but also for STING translocation away from the endoplasmic reticulum following DNA stimulation (Almine et al., 2017). Moreover, both studies suggest that the function of interferon-inducible p200 family members may be dependent on the cell type and situation under which the activity of cGAS/cGAMP/STING/TBK1/IRF3 signalosome is initiated.

As a “stimulator of interferon genes,” STING also is a direct innate sensor of so-called vita-PAMPs, the signatures of bacteria viability (Sander et al., 2011; Mourao-Sa et al., 2013). Among these are c-di-AMP and c-di-GMP (functioning as a second messenger in bacteria but not in mammals) and bacterial messenger RNA. Cyclic dinucleotides are involved in complex biological processes, such as biofilm formation, virulence, and photosynthesis. The original publications on these subjects demonstrated the recognition of c-di-AMP and c-di-GMP by STING, thereby initiating the production of type I interferons through the TBK1/IRF3 axis (Burdette et al., 2011; Jin et al., 2011; Barker et al., 2013). This pathway of innate immune recognition cannot be generally applied, however, in the framework of *Francisella* infection models. Only the genome of *F. novicida* encodes *F. novicida*-specific genes controlling production and degradation of c-di-GMP, and these genes are not present in *F. tularensis* LVS or *F. tularensis* SchuS4 strain genomes (Zogaj et al., 2012). We have no data on possible recognition of other *Francisella species' or strains'* vita-PAMPs, and thus we cannot exclude the possibility that STING really operates as a cell sensor in *Francisella* infection models. It has been proven, however, that STING is needed for early (8 h p.i.) but not late (24 h p.i.) IFN β production in an *in vivo F. tularensis* LVS model (Jin et al., 2011). The role of STING in the *Francisella* infection model therefore seems to be unambiguously proven, but the signaling pathway is still to be clarified. The late IFN β production, as has been mentioned, was shown to be a STING-independent process, and the multilevel, spatiotemporal character of the innate immune recognition process has been documented repeatedly.

Collectively, all these data from the literature may suggest that the recognition of *Francisella* inside the host cell is a dynamic process depending on the condition of the *Francisella* itself, host cell type, and influence of factors of the microenvironment within which the detection occurs. It seems critical for deciphering the complexity of innate response processes to understand first the signaling processes leading to discrimination between PAMPs/DAMPs and vita-PAMPs during early stages as bacteria invade naïve as well as epigenetically transformed host cells. Once fully understood, such processes may consequently be implemented into real *in vivo* conditions occurring within a defined time interval.

### Cell Autonomous Defense Mechanisms Contribute to Innate Immune Recognition

Recognition of pathogens by PRRs activates the cell surveillance pathways that orchestrate the cell responses to resolve a specific bacterial insult at the single cell level. Constitutive cell-autonomous immunity based on preexisting processes responds quickly due to the preexisting molecular and structural bonds. The IFN-inducible mechanisms of cell-autonomous defense, however, are rather the consequence of primary PAMPs recognition by cell compartment-specific PRRs and are dependent on the activation of IFN-inducible genes. Therefore, their response is delayed. The two types of cell-autonomous defense system are interconnected and collectively comprise a cell surveillance system.

The system of innate immune cell receptors detects the initial phases of host–pathogen recognition. The molecular interactions between host and microbe damage cell integrity and activate the cell-autonomous stress response. Engagement of PRRs in recognition of PAMPs or DAMPs is one of the processes that initiate the cellular stresses. It is not surprising, therefore, that stress sensors are involved in the signaling pathways leading to induction or modification of innate immune responses. Signaling from PRRs with parallel accumulation of misfolded or inappropriately post–translationally modified proteins in the endoplasmic reticulum (ER) triggers a group of conserved emergency rescue pathways known as unfolded protein response (UPR) (Walter and Ron, 2011; Gardner et al., 2013; Hetz and Papa, 2018).

*F. tularensis* strain LVS has been shown to trigger rapid deglycosylation of host membrane proteins (Barel et al., 2012) due to the expression of enzymes producing *N*- and *O*-linked glycosylation (Barel et al., 2016). An important effect related to intracellular existence of *Francisella* is an increased expression of GRP78/BiP (Barel et al., 2016), which is an ER stress chaperone required for proper folding and assembly of newly synthetized proteins (Lee, 2005; Luo et al., 2006). In general, the expression of glycosylated GRP78/BiP is followed by activation of IRE1 (Barel et al., 2016) through a binding/release mechanism. IRE1 is an ER-transmembrane protein important for sensing and responding to misfolded protein-GRP78/BiP dissociation and is one of three recently known arms of UPR (Hetz and Papa, 2018). Upon activation by misfolded proteins, IRE1 oligomerizes and initiates the unconventional splicing of the transcription factor XBP1 mRNA and leads to translation of the functional transcription factor (Calfon et al., 2002; Ghosh et al., 2014). *Francisella*, moreover, modulates the activation of the other two arms of UPR. While decreasing PERK (protein kinase RNA-like endoplasmic reticulum kinase) phosphorylation, this induces slight expression of the cleaved active form of ATF6 (Barel et al., 2016). The sequences of signaling events induced by *Francisella* inside the individual cell associated with so-called ER stress may be the cell-autonomous defense mechanisms designed to eliminate cell damage rather than to eliminate invading bacteria (Pillich et al., 2016).

**Figure 2:**
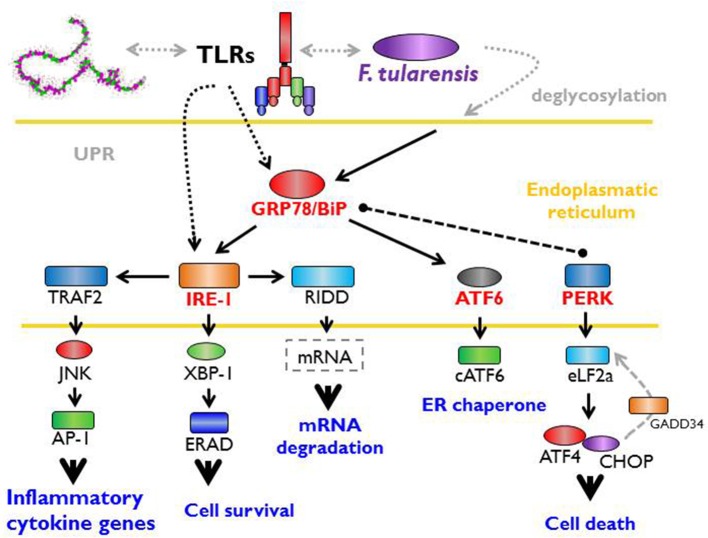
Unfolded protein response, ER stress, and *Francisella*. The interaction between host and microbe damages cell integrity and activates the cell autonomous stress response. Signaling from PRRs (pattern recognition receptors) with parallel accumulation of misfolded or inappropriately post-translationally modified proteins in the endoplasmic reticulum (ER) triggers so called unfolded protein response (UPR). This important effect related to the intracellular existence of *Francisella* increases the expression of GRP78/BiP chaperone (binding immunoglobulin protein) required for proper folding of newly synthetized proteins and lead to the activation of IRE-1 (inositol-requiring enzyme 1). Moreover, *Francisella* modulates the activation of other two arms of UPR via activating transcription factor six and inhibition of protein kinase RNA-like endoplasmic reticulum kinase). Signaling events induce by *Francisella* associated with ER stress may be considered as a cell-autonomous defense mechanisms designated to the elimination of cell damage rather that to eliminate bacteria.

The interconnection of PRRs recognition of PAMPs and a cell-autonomous defense response may be effectively demonstrated by the fact that sensing of *Francisella* by TLRs specifically activates one arm of UPR (IRE1-XBP1 signaling module activation), an effect that requires proximal TLR signaling (Martinon et al., 2010). It is important to note that the TLR-dependent IRE1-XBP1 signaling module activation occurs in this case without any signs of ER stress response and, as such, may not necessarily be related to ER stress *per se* (Martinon et al., 2010). The data from the *Francisella* experimental model thus document direct participation of signals originated from ER engagement on multichannel processes leading to effective recognition of invaded *Francisellae* (Figure 2).

Interconnection among the innate immune recognition affected by PRRs and ER stress-induced cellular defense responses can be even more complex. In general, ER stress response is directly linked with induction of autophagy and initiation of mitochondrial stress (Kaufman and Malhotra, 2014; Bronner et al., 2015). Both these processes are considered to be regulators of innate immune responses (Levine et al., 2011; Moretti and Blander, 2017). Stressed ER induces release of Ca^2+^ from ER stores that are accepted by mitochondria and constitute a signal to mitochondria that results in calcium overload generating mitochondrial depolarization and production of reactive oxygen species. The mitochondrial reactive oxygen species (mtROS) has been shown to activate signals through the JNK, p38, and/or ERK signaling pathways (Bulua et al., 2011) along with the processes leading to inflammasome activation. These conclusions are based on model studies with mitophagy/autophagy blockade, which leads in turn to mtROS generation and activation of NLRP3 inflammasome (Nakahira et al., 2011; Zhou et al., 2011). Concerning a real infection model using *Francisella* strains, the mtROS has been shown to be required for optimal activation of the AIM2 inflammasome (Crane et al., 2014). In parallel, cytosolic Ca^2+^, as a consequence of ER stress, initiates the signaling through CaMKK2 activated by Ca^2+^-calmodulin complex and downstream targets CaM kinases and AMPK (Hurley et al., 2005; Green et al., 2011). The phosphorylation of CaMK1, which is one of the three CaM kinase types, and phosphorylation of AMP kinase comprise one of the late events after bone marrow-derived dendritic cells are infected by *Francisella* strains (Fabrik et al., 2018).

**Figure 3:**
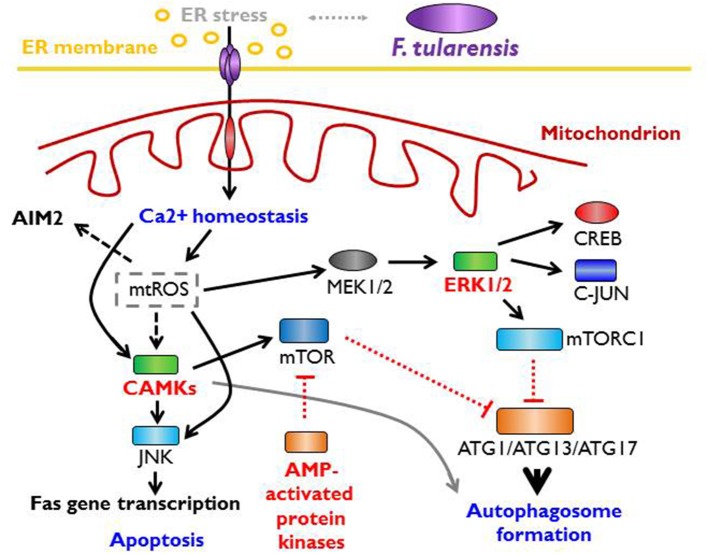
Mitochondrial stress signaling and *Francisella*. Endoplasmatic (ER) stress is directly linked to autophagy and the induction of mitochondrial stress response. Stressed ER releases Ca^2+^ ions that are accepted by mitochondria and results in calcium overload degenerating mitochondrial depolarization and production of mitochondrial reactive oxygen species (mtROS). This processes lead to activation of AIM2 inflammasome and the activation of signals via CAMKs (calmodulin-dependent protein kinases) that leads to the activation of apoptotic pathways or the activation of mTOR (mammalian target of rapamycin), master regulator of cell growth, metabolism, and the critical component of signaling network.

One of the targets of CaMKK-activated AMPK is mTORC1, which subsequently activates mTOR-dependent autophagy (Høyer-Hansen et al., 2007). mTOR (mammalian target of rapamycin), as a central sensor of multiprotein complex mTORC1, is considered to be a master regulator of cell growth and metabolism (Laplante and Sabatini, 2009, 2012, 2013) as well as a critical component of a complex signaling network in the context of innate immunity (Abdel-Nour et al., 2014; Saleiro and Platanias, 2015; Weichhart et al., 2015; Jones and Pearce, 2017; Linke et al., 2017; Moretti and Blander, 2017). Thus, the main metabolic and functional results of this global signaling network connecting the cell-autonomous defense can be the initiation of intercellular signaling, which epigenetically and functionally reprogrammed the cells of the immunoregulatory system, and the reprograming of the signaling cell itself based on the type of signaling cascades outcome in the form of preserving cell integrity (homeostasis, using the autophagy, ER-phagy or mitophagy processes) or induction of some type of programmed cell death mediated by an intracellular program (Figure 3).

The initiation of sequential signals after mutual interaction of *Francisella* with a host cell may reflect the direct or indirect interaction with the cellular organelles during initiation of entry into the host cell or in the course of intracellular trafficking of the bacterium. Starting with the interaction of *Francisella*-containing phagosome with endosomal membranes and through induction of a spliced form of XBP1 transcription factor, the participation of ER stress in the early stages of host–pathogen interaction is demonstrated. The contribution of cell autonomous defense mechanisms seems to be a critical event during innate immune recognition. Generation of mtROS by targeted mitochondria may suggest the interaction of *Francisella* with cell-autonomous stress responses in a spatiotemporal arrangement. *Francisella* is not the only bacterium that initiates the cell-autonomous stress responses after host cell infection. Intracellular pathogens like *Brucella, Legionella*, or *Toxoplasma*, as well as viruses, require extensive interactions with intracellular membranous compartments to initiate successful replication of bacteria. That very replication, meanwhile, enables their successful recognition and the initiation of immune responses (Roy et al., 2006; Janssens et al., 2014).

Despite all the progress made through analyzing experimental models, it still is not entirely clear how the signaling systems of PRRs and cell-autonomous stress responses are interconnected. Helpful in this respect can be the consideration of two interconnected cellular self-defense mechanisms categorized as a constitutive cell-autonomous immunity and IFN-inducible cell-autonomous defense. The first mobilizes preexisting processes; the latter is dependent on the activation of IFN-inducible genes and autocrine (in later stages of infection also paracrine) effect of proteins coded by them. The cell-autonomous effector mechanisms have evolutionarily ancient roots and were gradually specialized for the defense of specific cell (sub)types (MacMicking, 2012; Randow et al., 2013; Gaudet et al., 2016). It is possible to assume, however, that the STING molecule is an integrating link in the chain of events. STING had initially been characterized as an essential signaling adaptor transmembrane protein localized at ER membranes which was indispensable to type 1 IFN production (Ishikawa and Barber, 2008; Ishikawa et al., 2009; Gründler et al., 2013). STING, as an endoplasmic reticulum resident membrane protein that is partially localized on mitochondria and mitochondria-associated membranes to be activated, translocates from the ER toward the Golgi compartment to recruit TBK1. STING has been shown to be the mediator of ER stress upon the recognition of invading Gram-positive bacteria by c-di-AMP as a vita-PAMP (Moretti et al., 2017). Those authors speculate that activation of STING is accompanied by its conformational changes that might disrupt the process of protein folding in the ER lumen, or, alternatively, STING activation might be an indirect signal for ER stress sensor phosphorylation (Moretti and Blander, 2018).

We can also add that upon activation STING relocalizes from the ER area through the Golgi complex to perinuclear regions via a mechanism resembling non-canonical autophagy (Ishikawa et al., 2009). That may in itself be a signal for cell-autonomous stress responses. Such an arrangement can be realized when *Francisella* resides inside the cell. *Francisella* has repeatedly been demonstrated to be enclosed within a multi-membranous compartment (Checroun et al., 2006; Hrstka et al., 2007) by the process characterized as Atg5-independent non-canonical autophagy (Steele et al., 2013). The enclosure of *Francisella* into autophagosome, however, is not generally characteristic of *Francisella's* intracellular trafficking. The process of autophagosome induction is dependent on the infected cell types (Akimana et al., 2010) and virulence of the invading strains (Santic et al., 2009; Ramond et al., 2015). Although autophagy is generally regarded as one of the intracellular defense mechanisms, some authors consider autophagy in the case of *Francisella* as a source of nutrition (Steele et al., 2013). It is very likely that only replication-impaired strains are cleared by autophagy/xenophagy and that the replication-competent bacteria resist autophagic recognition (Chong et al., 2012; Barel et al., 2015). Such general processes as reticulocyte maturation or pluripotent stem cells metabolic reprogramming utilize Atg5-independent non-canonical autophagy for mitochondrial clearance (Zhang et al., 2009; Ma et al., 2015). It is perhaps justified to speculate that recognition of active mitochondria that produce mtROS by invaded *Francisellae* may induce the target process to eliminate mitochondria, which are themselves of bacterial origin. Capture of *Francisella* into autophagosomes could be an accidental effect, the dominant role being played by O-antigen at the surface of *Francisella* and which may regulate autophagy avoidance at the very beginning of *Francisella's* interaction with the host cell (Case et al., 2014; Härtlova et al., 2014).

### *Francisella* Interferes With Host Cell Defense

Having been exposed to different microenvironments during their interaction with host cells, intracellular bacterial pathogens have developed mechanisms to control their entry into the host cell and subsequently the contacts of the bacterium-containing phagosome with the host endomembrane system of various cellular organelles. Either passively or actively, *Francisella* seems to manipulate all levels of the host cell defense, starting from manipulation of complement activation, to reaching the inner space of the host cell, and through to manipulating those signals determining the final fate of the infected cell.

In contrast to extracellular bacteria, *Francisella* needs to be internalized by host cells in order to complete its genetically programmed life cycle. Some data suggest the binding of C3 fragments, C4b protein, and regulator of complement activation factor H to *Francisella* surface components (Ben Nasr and Klimpel, 2008). The factor H, as a cofactor, degrades C3b to iC3b. That can contribute to complement resistance and initiate an opsonin-induced uptake by the host cell (Harrison and Lachmann, 1980). The signals originating from ligation of PRRs by *Francisella* components are modulated shortly after the initial host cell–*Francisella* contact. The signaling through TLRs seems to be downregulated by limited expression of CD14, which can limit the signaling through TLR2 and/or TLR4 (Butchar et al., 2008). Moreover, *Francisella* influences the assembly of TRAF6 and TRAF3 complexes that control the transcriptional responses of PRRs ligation by inhibition of K63-linked polyubiquitination, and that can further limit the pathogen recognition by these pathways (Putzova et al., 2017).

Another example of *Francisella's* ability to interfere with host cell defense can be seen in suppression of the PI3K/Akt1 signaling pathway (Butchar et al., 2008) through manipulating inositol phosphatase SHIP activation (Parsa et al., 2006; Rajaram et al., 2009) or by increasing the expression level of the Akt antagonist PTEN (Melillo et al., 2010). Contrary to the SHIP and PTEN regulatory effect on PI3K/Akt signaling the downregulation of MyD88, and in parallel downregulation of SHIP-1, as a consequence of miR-155 expression after *Francisella* infection of monocytes/macrophages (Cremer et al., 2009), might be the “closing” signal for subsequent ligation of PRRs on the same cell rather than regulation of the initial signaling of innate immune recognition of pathogen (Cremer et al., 2012; Bandyopadhyay et al., 2014). Subversion of the MAP kinases signaling pathways, along with the downregulation of Akt signaling, has several times been demonstrated using different *Francisella* models (Telepnev et al., 2005; Huang et al., 2010; Medina et al., 2010; Dai et al., 2013).

It should be noted that the profile of signaling pathways activation or downregulation demonstrated in different experimental systems is dependent on the stage of host cell–*Francisella* interaction and is never absolute. Immediate activation of signals after initial interaction is demonstrably needed for *Francisella* entry into host cell; after internalization, the modulation of signaling is targeted to eliminating the autonomous host cell's defense system and to manipulating the host cell's ultimate fate in order to ensure realization of the bacterium's genetically programed cell cycle. Such signal reprograming may be generated by PRRs crosstalk during outside-in signaling. The crosstalk between CR3 and TLR2 during highly virulent *Francisella*-human monocyte-derived macrophages interaction downregulates TLR2-dependent pro-inflammatory responses by inhibiting MAPK activation (Dai et al., 2013). In this manner all other intercellular communications are modulated in the framework of innate as well as acquired immune responses.

To complete the list of *Francisella's* signaling pathways subversions, the protein coded by gene locus *FTL_0325* may provide an example of subverting the NFκB signaling. The OmpA-like protein FTL_0325 and its ortholog FTT0831c are initiators of the delay in pyroptotic cell death of infected macrophages during the early stages of infection. FTL_0325 impacts proIL-1β expression as early as 2 h post infection and delays activation inflammasomes in a TLR2-dependent fashion (Dotson et al., 2013). Both proteins mediate immune subversion by interfering with NF-κB signaling (Mahawar et al., 2012). Moreover, the ortholog FTT0831c of *F. tularensis* subsp. *tularensis* inhibits NF-κB activity primarily by preventing nuclear translocation of the p65 subunit (Mahawar et al., 2012). The interference of *Francisella* structural or secreted components with host cell signaling pathways is substantially sufficient to influence the final fate of *Francisella*-infected host cell.

ROSs, and especially mtROS, have, along with the effector defense function, positions in the signaling pathways contributing to expression of autophagy (xenophagy) processes and/or induction of infected cell apoptosis. To subvert ROS production in neutrophils, *Francisella* has been shown to block either the assembly of the NADPH oxidase integral membrane gp91^phox^/p22^phox^ components or the phosphorylation recruitment of its cytosolic p47^phox^/p40^phox^ subunits (McCaffrey et al., 2010). AcpA interacts directly with NADPH oxidase components and blocks complex assembly (Mohapatra et al., 2010). Moreover, such enzymes as superoxide dismutase (Bakshi et al., 2006), catalase (Rodionova, 1976), and other acid phosphatases (Mohapatra et al., 2010) contribute to eliminating oxidative bursts in neutrophils and elsewhere. Whether or not these bacterial enzymes are able to eliminate both the NADPH-induced ROS as well as mtROS is not yet clear.

Due to the regulation of PI3K/Akt and MAPK signaling pathways and/or modulation of SHIP and PTEN regulatory effect, *Francisella* controls the type of host cell death. Different *Francisella* experimental models have demonstrated different types of cell death as a consequence of *Francisella* infection. Apoptosis of *Francisella*-infected cells has been recognized in *in vitro* as well as *in vivo* experimental systems (Lai et al., 2001; Lai and Sjöstedt, 2003; Wickstrum et al., 2009; Lindgren et al., 2013). Host cell death in the presented models was affected through activation of a caspase-3-dependent mechanism and not of a caspase-1-dependent one. In this case, therefore, apoptosis predominated over pyroptosis in eliminating interacted cells. Pyroptosis, which has been redefined as gasdermin-mediated programmed necrosis, is initiated by inflammasome assembly, dependent upon activation of caspase-1, and accompanied by IL-1β and IL-18 production, was demonstrated as a result of murine elicited macrophage infection by *F. novicida* (Mariathasan et al., 2005). Gasdermin D, as a specific substrate of inflammatory caspases with pore-forming activity of its N-terminal cleavage product (Liu et al., 2016), has been shown to participate in the regulation of IFNβ production by depletion of intracytosolic potassium via forming of membrane pores, which is a signal sufficient to inhibit c-GAS-dependent signaling leading to IFNβ production (Banerjee et al., 2018). The imbalance of ions and energy depletion by *Francisella* manipulation of mitochondria can lead to mitochondrial functional collapse followed by oncosis at later time points of cell infection (Jessop et al., 2018). In contrast to what is seen in macrophages, *F. tularensis* inhibits Bax translocation to neutrophils' mitochondria. That is a critical step for mitochondrial stabilization, which, downstream, limits neutrophil apoptosis (McCracken et al., 2016). Manipulation of autonomous cell defense systems modulates the phagosome biogenesis and/or phagosomal escape or it is required for proliferation within the cytosol. Some modulatory events could interfere with host cell transcription events, could regulate the host cell cycle or, in general, could also manipulate the evolutionarily conserved eukaryotic regulatory processes of arthropod vectors and mammalian cells (Akimana et al., 2010; Akimana and Kwaik, 2011). The manipulation of host cell signaling pathways by *F. tularensis* thus dictates the final fate of both the infected cell and *Francisella*.

## Spatiotemporal Concept of Innate Immune Recognition

The morbidity and mortality of infection caused by different *F. tularensis* strains vary also according to the gateway of infection and thereby demonstrate an example of relative bacterial virulence (Conlan et al., 2011). This phenomenon can be attributed to the response of the specific cell subtype with which the *Francisella* interacts primarily at the original site of infection. Alternatively, or subsequently, the relative virulence of *Francisella* can be explained by spatiotemporal effect of intercellular communication during innate immune recognition and expression of integrated signals to activate the adaptive immune responses. We have presented recently the concept of innate immune recognition based on so-called signaling windows (Krocova et al., 2017). The basic idea is that there exist functional cellular immune response modules that temporarily, and in a spatiotemporal configuration, regulate innate immune recognition and sequentially modulate induction, regulation, and expression of the adaptive immune response. We had hypothesized that cytokine messages produced by primarily infected cells modulate the functional profile of a secondarily reacting cell. The type of cytokine message will be dependent upon the cell type or subtype that initiates the innate recognition. What could be termed the “quality” of the innate immune recognition will be further dependent upon the ability of the cells that will interact with the bacterium in secondary order to receive the cytokine message. This means that it will be dependent upon the spectrum of surface receptors able to recognize the cytokine messages and simultaneously (or subsequently) recognize the incoming bacterium. The cytokine response of such cell—and it does not matter whether it is of the same or a different cell type—would necessarily be different from the message produced by the originally infected cell. Moreover, antigen-presenting cells reacting in the secondary order might have quite different processes for recognizing, handling, and processing a bacterium than do the primary infected cells. Cellular hosts thus create a four-dimensional signaling network in the host organism. The impact of modulated functional processes of cells at the specific microenvironment in relation to invading microbes could have a profound impact on expression of the adaptive immune response (for more detail, see Krocova et al. (2017)).

The dominant role in triggering and streamlining of innate immune response is played by the characteristics of innate immune recognition followed by epigenetic reprogramming of innate immune cells, which create the hierarchy of immune response functional modules. This phase of immune response induction is critical for inducing and regulating the expression of adaptive immune response. Sometimes contradictory data from infection models make it difficult to construct a unifying concept of processes that affects the innate immune recognition of intracellular pathogens. Some *in vitro* studies provide evidence that *F. tularensis* LVS represses inflammasome activation, while other data demonstrate that *F. tularensis* LVS increases mRNA levels of proinflammatory cytokines and that this is followed by increased protein secretion. These studies, however, frequently reflect different spatiotemporal dimensions. It should be emphasized that the virulence of a bacterium on the one hand and the resistance of host cell(s) on the other hand are dictated by what may be termed the “historical memory” of both, and both mutually generate at any given time a microenvironment affecting all subsequent events in the induction of immune responses. Thus, the innate immune recognition of intracellular bacterial pathogen(s) is a multistep process that is dependent on the modulation of epigenetic reprograming of innate immune cells by a microenvironment that is changing in time.

## Conclusion and Perspectives

We do not yet fully understand the innate immune recognition processes leading to the induction of adaptive immune response. In addition to imperfect knowledge as to the pathogenesis of intracellular bacteria, another main reason for why we so far have failed to develop an effective vaccine against tularemia lies in our lack of understanding of innate immune recognition processes. It can be said very simply that this puzzle will remain uncompleted unless and until we develop sufficient chronological information from dynamic studies of signaling pathways activation that will explain to us the logic of interplay among various cell (sub)types. Based on the data from different *Francisella* models, we have presented here the scheme of fundamental signaling pathways activated and modulated by interaction of a host cell with *Francisella* and a brief summary of innate immune recognition processes respecting its multifactorial character.

Let us quote from a 1946 lecture by Professor Jan Belehradek: “In nature there are no living systems other than integral and inseparable organisms and their associations to higher entities. Organisms can be broken down into parts, but always something is missing when they are separated into these parts. What is missing is just the integrity. The parts are created by abstract resolution, but they do not express correctly or adequately what caused everything to be formed into one whole.” This is just the problem of the data from *in vitro* systems, because “always something is missing.” Moreover, we still accept a very simple model that assumes intracellular parasitism constitutes searching for a food supply (Santic and Abu Kwaik, 2013). The interaction of intracellular bacteria with membranous compartments inside the host cell, including the mitochondria having bacterial origin, may, however, indicate that the processes inside the host eukaryotic cell might be the impulse for further evolution of the pathogen and probably its host. The demonstrated positive selection of some *Francisella* genes may confer some evolutionary advantage to the bacterium (Gunnell et al., 2016). Studies oriented to mutual interaction of cellular organelles with *Francisella* containing vacuole and *Francisella* itself could provide valuable information allowing us to understand the interrelationships during the host–pathogen interaction.

## Author Contributions

All authors listed have made a substantial, direct and intellectual contribution to the work, and approved it for publication.

### Conflict of Interest Statement

The authors declare that the research was conducted in the absence of any commercial or financial relationships that could be construed as a potential conflict of interest.

## Abbreviations

AIM2: absent in melanoma 2
BGPs: guanylate-binding proteins
*F*. *tularensis*: *Francisella tularensis*
FPI: *Francisella* pathogenicity island
IRGBs: immunity-related GTPases
LPS: lipopolysaccharide
LVS: live vaccine strain
mtROS: mitochondrial reactive oxygen species
NLRs: leucine-rich repeat-containing protein receptors
PAMPs: pathogen associated molecular patterns
PRRs: pattern recognition receptors
TLRs: Toll-like receptors
TOR: mammalian target of rapamycin.
